# Predicting the spread of the Asian hornet (*Vespa velutina*) following its incursion into Great Britain

**DOI:** 10.1038/s41598-017-06212-0

**Published:** 2017-07-24

**Authors:** Matt J. Keeling, Daniel N. Franklin, Samik Datta, Mike A. Brown, Giles E. Budge

**Affiliations:** 1 Zeeman Institute: SBIDER, University of Warwick, Coventry, CV4 8UW UK; 2 Warwick Mathematics Institute, University of Warwick, Coventry, CV4 8UW UK; 3 School of Life Sciences, University of Warwick, Coventry, CV4 8UW UK; 4 Animal and Plant Health Agency, Sand Hutton, York, YO41 1LZ UK; 5 Institute for Agri-Food Research and Innovation, Newcastle University, Newcastle upon Tyne, Tyne and Wear NE1 7RU UK; 6 Fera, Sand Hutton, York, YO41 1LZ UK

## Abstract

The yellow-legged or Asian hornet (*Vespa velutina*) is native to South-East Asia, and is a voracious predator of pollinating insects including honey bees. Since its accidental introduction into South-Western France in 2004, *V. velutina* has spread to much of western Europe. The presence of *V. velutina* in Great Britain was first confirmed in September 2016. The likely dynamics following an initial incursion are uncertain, especially the risk of continued spread, and the likely success of control measures. Here we extrapolate from the situation in France to quantify the potential invasion of *V. velutina* in Great Britain. We find that, without control, *V. velutina* could colonise the British mainland rapidly, depending upon how the Asian hornet responds to the colder climate in Britain compared to France. The announcement that a second hornet had been discovered in Somerset, increases the chance that the invasion first occurred before 2016. We therefore consider the likely site of first invasion and the probabilistic position of additional founding nests in late 2016 and early 2017. Given the potential dispersion of *V. velutina*, we conclude that vigilance is required over a large area to prevent the establishment of this threat to the pollinator population.

## Introduction

The yellow-legged or Asian hornet (*Vespa velutina nigrothorax*) is native to much of Asia, from north-eastern India throughout southern and central China as far as Taiwan and as far south as Indonesia^1^. In its native environment, it hunts a range of pollinating insects, including the Eastern honey bee (*Apis cerana*), hovering outside of colonies and catching foraging bees as they return to the hive, a behaviour known as hawking. The Eastern honey bee has developed natural avoidance behaviours to combat hawking, such that a strong colony can withstand the impacts of this type of predation^2, 3^.

It is believed that *V. velutina* queens first arrived in Europe in 2004, imported with Chinese pottery into the Lot-et-Garonne regions of South West France^4, 5^. From this single point of invasion the Asian hornet has since spread rapidly to invade Spain in 2010^6^, Belgium and Portugal in 2011^7, 8^, and Italy in 2013^9^ (for complete map see ref. 10). *V. velutina* nests are founded by a single mated queen which has overwintered, known as a foundress. At the end of the summer, each successful nest will produce multiple foundresses, which are mated and hibernate over winter while the rest of the colony dies. The following spring, foundresses emerge and disperse to form a primary nest within which to raise the first workers. The scale and speed of the European invasion is thought to have been assisted by the ability of foundress queens to disperse over long distances (potentially exacerbated by human movements of hibernating queens); recent work on *V. velutina* in France assumed a wave speed of around 70–80 km/year^11, 12^.

In Europe, *V. velutina* predates upon many important pollinators including hoverflies and bumblebees, but more than half its diet comprises the Western or European honey bee (*Apis mellifera*)^4, 5^. European honey bees have been shown to mount an inefficient and disorganised defence to *V. velutina* hawking, and entire colonies can be destroyed by predation^3^. This potentially places serious pressures on European honey bee populations that are already under considerable stress^13^.

*V. velutina* was first confirmed in Great Britain in September 2016, when worker hornets were seen foraging at an apiary near Tetbury, Gloucestershire. The nest was later found and destroyed, and an extensive search of the surrounding landscape conducted without finding any additional nests or signs of *V. velutina* activity. Subsequently a second Asian hornet was reported in North Somerset. Genetic analyses, by the National Bee Unit, suggest both hornets share the same haplotype as the mainland European population but could be related no nearer than the grandparental generation. It remains unclear whether *V. velutina* established nests prior to 2016 or whether these represent independent incursions from a common source in Europe. However, in view of this threat, there is an immediate need for reliable methods capable of predicting its onward spread and directing eradication attempts if this incursion is to be contained.

Mathematical models, matched to available data, are useful tools for forecasting invasion and establishment patterns, and determining the likelihood of success for control and eradication policies^14–16^. However, the population spread and growth of *V. velutina* remains poorly defined, in part due to a lack of detailed knowledge of the insect’s natural history and sparse field observations in the European context. Yet this knowledge is key to refining eradication strategies and determining likely spread of localized invasions. Observations in terms of detection and destruction of *V. velutina* nests from the Andernos-les-Bains region of southwest France (around 100 km from the initial site of invasion) provide the most detailed description of local *V. velutina* colonization following its invasion into Europe in 2004^17^. These data have been previously used to determine a simple mechanistic model of nest density, together with probabilities of detection whose success increases through time^18^. This density model, together with the observation that *V. velutina* are invading with an estimated wave-speed of between 67 and 82 km per year^11, 19^, enables us to develop a predictive model for Great Britain. This model captures the density-dependent and latitude-dependent production of the next generation of founder queens from each nest, the long-range dispersal of these new queens, and the suitability of the environment for new nests. As such, the model allows us to predict the stochastic likelihood of establishment following initial invasion and the impact of plausible control measures. Here we outline the formulation of this mathematical model and the associated parameter uncertainties, before focusing on the predicted spread with and without control measures.

## Methods

The full mathematical model constructed to simulate the spread of *V. velutina* is described in the Supplementary Material. The model stochastically simulates the potential establishment of *V. velutina* nests across the landscape of Great Britain, capturing the two processes of: generation of new queens from a nest, and dispersal of these queens across a heterogeneous landscape. Parameters are based on empirical evidence obtained from observations in Andernos-les-Bains^18^, although extrapolated to a UK setting by accounting for the climatic impact of latitude (Supplementary Material).

Each year the number of new queens per nest (that will successfully survive hibernation to produce a new nest) is assumed to be Poisson distributed with a mean that reflects the strength of local density-dependence, the suitability of the local environment, and the effect of latitude. The effects of density-dependent competition and the reproduction potential in the absence of density dependence are inferred by matching to eight years of observations in the Andernos-les-Bains region (Supplementary Material). We parsimoniously assume a linear decrease in the mean number of queens per nest with latitude, such that reproductive success drops to zero in northern England; this zero cline is in agreement with observations for the European hornet (*V. crabro*) and predictions from more detailed distribution modelling^20, 21^. While the linear assumption is inherently simple, there is little data to support this hypothesis, and we therefore consider throughout our uncertainty in the impact of latitude at the point of invasion.

Queens produced in one year disperse across the landscape, with the exponentially distributed mean dispersal distance of 28 km; this dispersal was determined to match reported invasive wave-speeds^11, 19^ (Supplementary Material). The choice of location for future nests is affected by the terrain type, with urban and agricultural areas preferred, capturing the recorded locations of nests from French national data^22, 23^.

Simulations begin with a single successful nest, and are iterated forward for 25 years to determine the stochastic range of dynamics. We seed the first nest in the Gloucestershire area of Great Britain, either placed near Tetbury, the same location as the first foraging *V. velutina* reported in Great Britain, or placed probabilistically at a location that is in agreement with invasion in 2015 and the detections in 2016. We either simulate the stochastic invasion dynamics unimpeded, or include probabilistic detection of nests. Eradication attempts are simulated using a fixed probability of nest detection, following which the nests are destroyed and so no foundresses are produced; those nests that remain undetected are free to produce new foundresses which disperse. These simulations are investigated in terms of how the probability of detection relates to the probability that an invasion will ultimately be eradicated. In addition, we include the strategy where local radial searches (of 2, 4, 8, 16 and 32 km) are performed once a nest is discovered, and assume these focussed searches have a high probability of detection.

## Results

**Figure 1:**
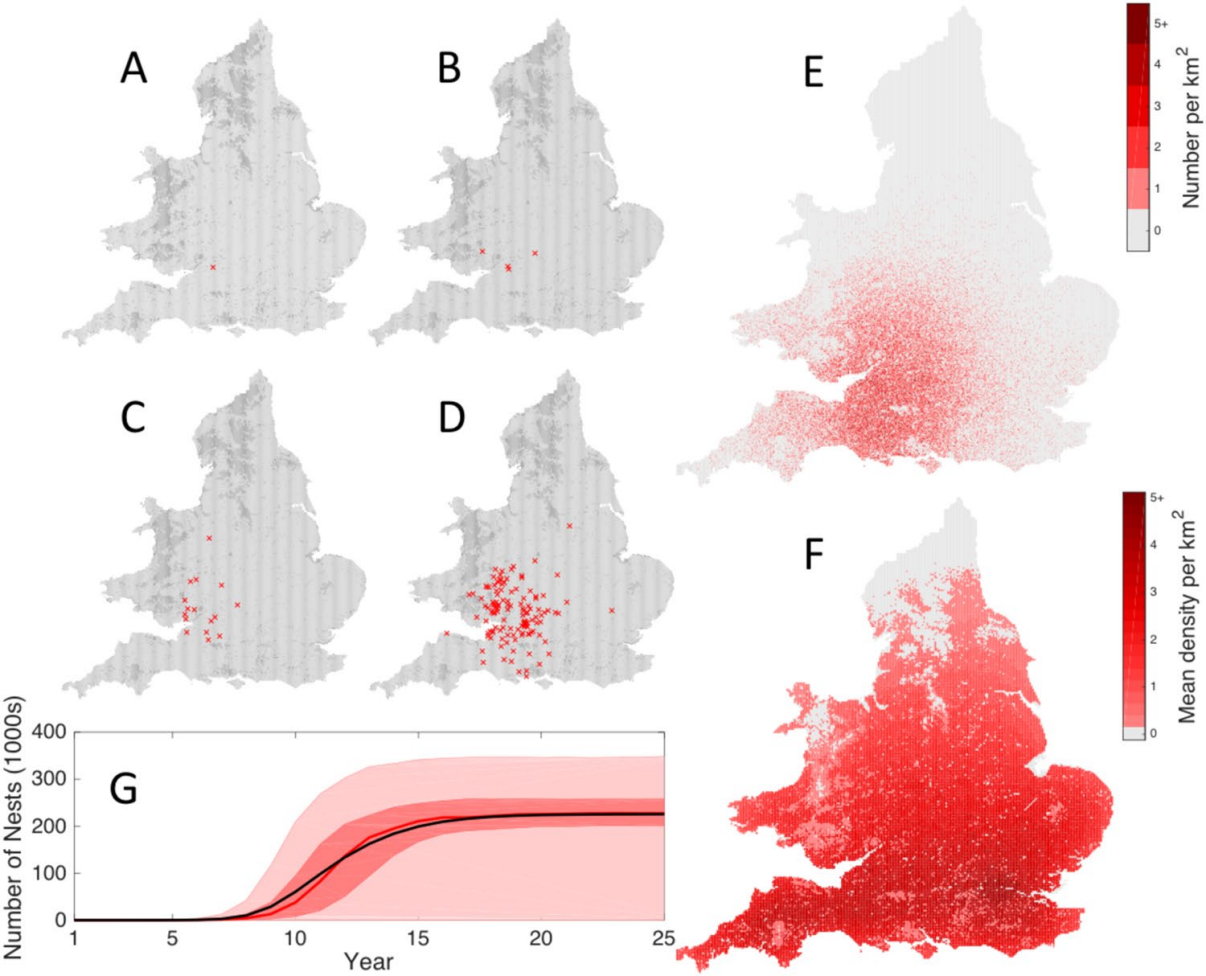
Dynamics of V. velutina nests in England and Wales showing its spatial spread and rapid increase in numbers. Maps A to E represent a single realisation of the spatial model with ecological parameters at the median of the MCMC posterior distributions based on the data from Andernos-les-Bains^18^. In maps A to D (which represent years 1, 2, 3 and 5) individual nest sites are marked with crosses, in map E (at 10 years) we show the density of nests in 1 km squares. Map F displays the long-term state (20 years following invasion), averaged across stochastic replicates and across parameter uncertainty. Graph G shows the temporal dynamics highlighting the mean (black), median (red) and 50% and 95% prediction intervals (shaded). (Map F and Graph G are from a thousand stochastic replicates with random draws from the posterior parameter distributions. Maps are generated from EEA Corine Land Cover data^28^ and displayed with bespoke software using Matlab^29^.)

The stochastic mechanistic model is first used to examine possible establishment and spread of *V. velutina* based on the discovery of an active nest near Tetbury, Gloucestershire. The simplest scenario is to examine the potential uncontrolled spread from this location, assuming that queens had already dispersed into the landscape before the nest was destroyed. Figure 1 provides a spatial representation of a single (successful) establishment. From a single nest (Fig. 1A) this realization produces 4 nests in the following year (Fig. 1B) which is slightly above the expected value, 16 nests by year 3 (Fig. 1C) and 129 nests by year 5 (Fig. 1D). After 10 years the invasion is predicted to be widespread, with over 50,000 nests in total and a high density of nests predicted in some regions, exceeding 5 nests per km^2^ (Fig. 1E). These simulations highlight the long-distance dispersal of *V. velutina* foundresses to form new nests, such that much of England and Wales can be colonized within a decade and carrying capacity is reached within 20 years – although parameter uncertainty and stochastic variability lead to huge variation between replicates (Fig. 1G). At carrying capacity (Fig. 1F) the impact of latitude and environment on the local abundance of nests become clearer; the early dynamics are, as expected, governed by dispersal and the expected number of successful foundresses per nest, whereas the long-term dynamics are governed by latitude and the suitability on the local habitat. The effects of latitude and the associated reproductive success is now explored in more detail in the Supplementary Material, and in the assessment of early invasion dynamics given below.

**Figure 2:**
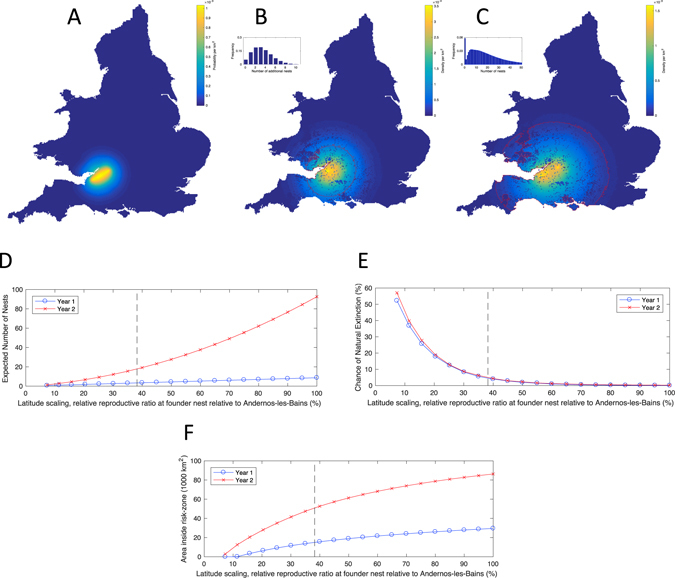
The likely spatial locations of nests in the years 2015, 2016 and 2017, and the sensitivity of results to the effect of latitude-on the numbers of nests. Given the two locations in which a nest and a hornet were found in 2016, map (**A**) shows the most likely positions of a common ancestor nest in 2015. Extrapolating forward from this probable location, maps (**B** and **C**) show the likely density of undiscovered nests in 2016 and new nests in 2017 respectively. The red contour is defined such that outside this (high-risk) region we would expect to find less than one nest. The inset histograms show the likely distribution of nests in each year, accounting for parameter uncertainty and stochastic variability. Graphs (**D**–**F**) explore the sensitivity of these findings to our assumption about the effects of latitude; in particular, we vary the linear function of latitude and plot on the x-axis the realised reproductive ratio at the likely location of the founder nest in 2015 relative to that inferred from the Andernos-les-Bains data (Supplementary Material). We show the expected number of nests (**D**), the chance that the invasion naturally dies out (**E**) and the area within the high-risk red contour (**F**) – blue lines correspond to Year 1 (2016), red lines to Year 2 (2017). (All results are calculated explicitly from the probabilistic rates in the Supplementary Material without having to use stochastic simulations. Maps are generated from EEA Corine Land Cover data^28^ and displayed with bespoke software using Matlab^29^.)

The announcement that a second *V. velutina* had previously been found some significant distance from the Tetbury nest, gives extra weight to the assumption that *V. velutina* may first have invaded in 2015, yet remained undetected. To investigate this possibility further, we perform three pieces of simple spatial analysis to determine: (1) the most likely location of an initial invasion in 2015; (2) over what range should we expect to observe nests in the year of discovery (2016), and how many remain undiscovered; and finally, (3) over what range we should expect to observe nests in the year following discovery (2017). Unsurprisingly, the most likely position of any invasion in 2015 is directly between the two sightings (Fig. 2A) although with some degree of spatial uncertainty. Given that we assume a Poisson distribution of new successful foundresses per nest, simple probabilistic rules mean that the detection and destruction of *V. velutina* nests does not change the predicted distribution of undiscovered nests. If we assume an initial nest in 2015, then our model suggests that on average an additional 3.3 daughter nests should be dispersed into the wider environment, with a high (96%) probability that at least one nest remains undiscovered. The spatial distribution of these potential nest locations is over a very wide area, although the focus remains around the Bristol area (Fig. 2B) – our high-risk zone is defined such that all but one nest are expected to lie within the red contour. Taking these predictions into 2017 (Fig. 2C), we find that, while the focal location remains, the probability of finding a nest increases in both spatial extent and in magnitude. By 2017 the mean predicted number of nests in the absence of further control is approximately 17, and from this large number the chance of stochastic extinction is extremely low.

Such predictions are naturally affected by the assumed reproductive potential at the point of invasion, which in turn depends upon our assumptions about the impact of latitude. We have parsimoniously assumed the reproductive potential drops linearly from the values estimated from Andernos-les-Bains to zero in the north of England; Fig. 2D–F explore this assumption in more detail. Unsurprisingly, both the number of expected nests and the size of the high-risk zone increase with the assumed reproductive ratio, while the chance of natural extinction reduces. All have a non-linear dependence. Our default assumption is that the UK founder nest has approximately 38% of reproductive potential of a nest in Andernos-les-Bains due to its more northerly latitude and therefore colder climate. Any scaling that reduces the reproductive potential below 8% of the value in Andernos-les-Bains leads to the rapid extinction of *V. velutina* from Great Britain.

**Figure 3:**
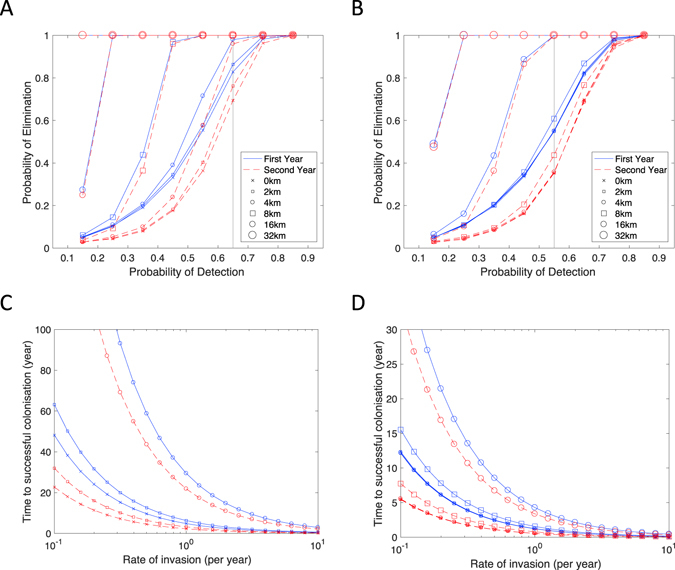
The probability of *V. velutina* eradication following successive years of nest detection and destruction. All results represent the average over (ten thousand) stochastic replicates and capture full parameter uncertainty. Solid blue lines represent simulations where detection (and destruction) begin in the year of invasion – when there is assumed to be only one active nest; dashed red lines correspond to simulations where the invading nest remained undiscovered in the first year, and probabilistic detection only begins in the second year. Crosses are for simulations without additional radial detection, while open symbols represent differing radii of detection. In (**A**) we assume that once a nest is probabilistically detected a thorough search of the area will find 99% of all nests within a given radius; in (**B**) we assume these radial searches only find 48% of nests (comparable to the detection probability in Andernos-les-Bains (Supplementary Material)). In (**C**) and (**D**) we investigate the expected time to successful colonisation when the UK is subjected to a constant rate of invasion; detection rates in (**C**) and (**D**) correspond to the vertical lines in (**A**) and (**B**). Search radii of 8 km or more in (**C**) and 32 km in (**D**) lead to permanent exclusion of *V. velutina*.

Given the huge damage that *V. velutina* can do to honey bee colonies and other important wild pollinators, an uncontrolled expansion would be disastrous, both in terms of the impact on the apicultural industry and the related decline in pollinator services. We therefore utilise our model to consider the impact of detection and destruction of nests in controlling a rapidly expanding invasion. We investigate the probability of eventual eradication for different probabilities of detection, and for different assumptions about the initial timing when probabilistic detection begins: in the first year (blue) or second year (red dashed) following invasion (Fig. 3). We assume that there is an independent probability of detecting (and subsequently destroying) each nest before it produces the next generation of founder queens. In practice this probability is the product of two independent probabilities: first detecting hornets feeding at an apiary, and second, discovering the nest by back-tracing the hornet flight paths. As anticipated, early initial awareness of nests (blue vs red) and/or high detection probabilities are required to achieve eradication, with detection probabilities in excess of 90% required to be confident of elimination. We note that this threshold represents an average over both stochastic realisations of the model and parameter uncertainty. The potential for the initial invasion to have occurred in 2015 (and hence for there to have been one year without control) would place the current invasion on the lower (red dashed) curves suggesting eradication would be more difficult.

As an additional element to this random detection of nests, we assume that once the presence of *V. velutina* is identified at a location, then localized radial searches will be conducted to find other nests. Assuming this can be done with great efficiency, such that 99% of all nests within the search radius are discovered, leads to substantial improvements in the chance of eradication (Fig. 3A). However, even with this intensive local searching, large radii are needed to ensure control if the primary chance of detection is low; this is because even in the early stages of an invasion nests can be widely dispersed. The findings from the Andernos-les-Bains region data (Supplementary Material) indicate that even when *V. velutina* are known to be in an area, only 48% (CI 33–61%) of nests are discovered while still active, such that their destruction halts the spread of *V. velutina*. When radial searches are reduced in efficacy to these values (Fig. 3B), their impact in controlling an outbreak is marginal except at the largest radii.

Invasion is unlikely to be an isolated incident. As the spread of *V. velutina* increases throughout mainland Europe, new invasions are set to become more likely. Mathematically, the control of these invasions can be treated independently, although in practice multiple invasions within a year may place additional stress on limited resources or lead to complacency once one nest is eradicated. Keeping with the independence assumption we extend the results to consider a stochastic rate of invasion and the expected time before a successful colonization. In both examples (Fig. 3C and D), limited local searching means that there is a finite (and often short) time until control efforts fail; however, with larger search radii (those not shown) permanent exclusion is possible.

## Discussion

Here we have developed a relatively parsimonious model for *V. velutina*, that is matched to eight years of observations from Andernos-les-Bains in France and extrapolated to the UK. Under the simplest extrapolation mechanism, which assumes the reproductive ratio decreases linearly with latitude, we have explored the invasive potential of *V. velutina* following invasion into the UK. We have shown that rapid nationwide colonization is possible (Fig. 1) and that even from a single invasive site the spatial distribution in subsequent years is potentially large (Fig. 2). This all means that a mixture of effective detection of nests together with effective radial searches is required if future invasions are to be controlled. One key sensitivity that we have explored is the reproductive ratio at the site of invasion relative to the value estimated in Andernos-les-Bains; unsurprisingly, this uncertainty plays a key role in our results and suggests that further entomological studies in northern Europe would be exceedingly helpful in precisely determining the risk to the UK. A time-series of observations from northern Europe comparable to that from Andernos-les-Bains would allow us to more robustly assess the impact of climate and therefore latitude. Better data on hunting behaviour of *V. velutina* in Europe would help to tighten the spatial aspects of density-dependent competition. While more comprehensive reports from across Europe would help to determine the long-range dispersal of this invader, only studies of *V. velutina* in the temperate maritime climate of Britain can truly inform a GB-centric model. Therefore, until such data are available our model remains a hypothetical construct, albeit based on the best data currently available.

Our results highlight that the methods for nest detection deployed in France provide inadequate control to affect eradication, with only 48% (CI 33–61%) of nests discovered. Given that successful eradication requires a high probability of early detection, policies could consider deploying technologies that improve nest detection. For example, radio tracking techniques developed to monitor insect foraging behavior^24^ could be used to tag foraging hornets and improve back-tracing to the nest location. Given that *V. velutina* have a preference for locating secondary nests in trees and maintain above ambient brood temperatures, sensitive forward looking infrared (FLIR) cameras^25^ could be deployed to ground teams or attached to unmanned aerial vehicles (UAVs; drones) to improve nest detection. In France, *V. velutina* have a preference for nesting in deciduous oak trees, allowing the large nests to be more clearly seen in the winter as the branches become bare. In the spring, foundress queen *V. velutina* can be baited into traps^26, 27^. Both methods could give rise to new locations from which to begin detection and eradication strategies.

The recent invasion of *V velutina* in England has highlighted Great Britain’s potential vulnerability to this predator despite its physical separation from mainland Europe. However, given the limited amount of data available there is considerable uncertainty surrounding the dynamics of *V. velutina* in Great Britain. We therefore speculate about a range of possible scenarios and what they imply for the likelihood of successful invasion and establishment. If the invasion first occurred in 2015, but no further Asian hornets are detected in 2017, then this clearly signals that the first nest probably only produced a limited number of queens and only one of these founded a viable nest (the one near Tetbury) which was subsequently destroyed. This would be reassuring as it would suggest that conditions in the south of England are not conducive to the establishment and rapid spread of *V. velutina*. However, if new nests are discovered in 2017 (and these are found to be daughters of the Tetbury nest) this gives added weight to the hypothesis that sustained invasion leading to establishment is possible.

Using this invasion and our simulation model, we have shown that without control it is possible for *V. velutina* to become established across most of England and Wales over 20 years; although this is highly dependent on assumptions concerning the impact of climate. In this case of rapid invasion, although moderate detection and destruction of nests will slow this spread, extremely high levels of detection are needed to achieve eradication (Fig. 3). Moreover, given the increasing numbers of *V. velutina* in mainland Europe, it is probable that multiple invasions will occur in the future, enhancing the need to detect with each invasion as quickly as possible. As the likely position of secondary nests can be far from the original source, this suggests that beekeepers over a wide area need to be vigilant if we are to prevent the establishment of *V. velutina*.

## Electronic supplementary material

Supplementary Material
