# Supplementary material for: Predicting the spread of the Asian hornet (Vespa velutina) following its incursion into Great Britain
Source: Sci Rep. 2017 Jul 24;7:6240. doi: 10.1038/s41598-017-06212-0 (PMC5524706; doi:10.1038/s41598-017-06212-0)
Supplement: Supplementary file 1 — Supplementary Material [file 41598_2017_6212_MOESM1_ESM.pdf]

Predicting the spread of the Asian hornet (*Vespa velutina*) following its incursion into Great Britain

Supplementary Material

Matt J. Keeling[1,2,3,] Daniel N. Franklin[1,3], Samik Datta[1,2], Mike A. Brown[4], Giles E. Budge[5,6]

[1] Zeeman Institute: SBIDER, University of Warwick, Coventry, CV4 8UW.
[2] Warwick Mathematics Institute, University of Warwick, Coventry, CV4 8UW.
[3] School of Life Sciences, University of Warwick, Coventry, CV4 8UW.
[4] Animal and Plant Health Agency, Sand Hutton, York, YO41 1LZ.
[5] Institute for Agri-Food Research and Innovation, Newcastle University, Newcastle upon Tyne, Tyne and Wear NE1 7RU.

[6] Fera, Sand Hutton, York, YO41 1LZ.

Here we extend the work in the main paper by providing more details of the observational data from Andernos-les-Bains, the mathematical model formulation, and the Bayesian parameter estimations.

OBSERVATIONAL DATA

Our data come from eight years of observations in the district of Andernos-les-Bains. Andernos-les-Bains is a small commune (just 20.1 km^2) in south-west France on the shore of the Arcachon Bay and only 100km from the estimated initial site of invasion of *V. velutina* into Europe in 2004.

The number of detected *V. velutina* nests in Andernos-les-Bains from 2007 to 2014 (Table S1) provides unprecedented detail on the invasion and growth of this species in a region. The data contain information on both smaller primary nests (which are established by queens after first emerging from hibernation and are then abandoned mid-season) and larger secondary nests, and whether these nests are active or abandoned when discovered. Only nests that are active when destroyed contribute to the control of this invader, as abandoned nests have served their purpose and the colony has moved on.

	Year							
	2007	2008	2009	2010	2011	2012	2013	2014
Active Primary	-	-	18	4	11	16	24	0
Active Secondary	-	-	14	18	22	34	39	79
Active Nests	1	12	32	22	33	50	63	79
Abandoned Nests	3	14	31	18	29	26	22	20

Table S1. Number of reported active primary and secondary nests, as well as abandoned nests detected in the Andernos-les-Bains region. For the first two years, the distinction of primary and secondary is absent.

This information is used to infer simple model parameters relating to a population with annual reproduction (i.e. new nests are generated each year) and with local density-dependence limiting reproduction as the local density of colonies becomes large.

MODEL FORMULATION

The model is a stochastic yearly time-step difference equation. A yearly time step is appropriate as it follows the life cycle of *V. velutina*; queens emerge from hibernation in early spring, build a small primary nest which is abandoned in the summer as the colony grows and a larger secondary nest built; in autumn, new queens are produced which disperse, after which the colony dies [1]. This type of rigid seasonal behaviour is mathematically simpler to capture using a discrete-time model. At each yearly step, the number and location of *V. velutina* secondary nests are calculated; hence this model steps from one summer to the next. In general, we seed simulations with a single invasive nest;

for simulations in Great Britain the location of this nest is chosen to match the detections that occurred in 2016.

Number of queens produced per nest. It has been reported that a single *V. velutina* invading foundress can establish a new viable population [2]. This is due to the high number of gynes a single female produces that succeed as queens themselves. The expected number of queens from a single nest (in the absence of density-dependence) that are successful in hibernating and starting their own colony is a measure of the reproductive potential, *r*. This quantity is estimated by matching a non-spatial version of the model to data from Andernos-les-Bains in France [3]. Results of this Bayesian parameter inference are given below.

The model assumes that each nest produces a Poisson distributed number of successful daughters that will generate new nests the following year. The mean of this Poisson distribution is reduced by localised density-dependent factors (conceptualised as local competition, C), the local environmental suitability, *E*, and the latitude of the nest (which is considered a proxy for a range of climatic effects that could influence success). This gives rise to a relatively simple stochastic discrete-time model for the number of nests in each year. On top of this reproductive process we add spatial dispersal of the new queens, and detection and destruction of the nests. The formalism for these processes is given below.

The strength of density-dependence experienced by nest i (C_i) is determined by a Gaussian competition kernel measuring the strength of interaction with all other nests in the population. Assuming, Δ_{ij} is the distance between nests i and j, and σ_c is a measure of local interaction distances, then the strength of competition is given by

$$C_i = \sum_{j=1}^{n} exp\left(-\frac{\Delta_{ij}^2}{2\sigma_c^2}\right). \quad (1)$$

This is effectively a measure of how likely two nests are to interact, thought of as competition over the same food source, where foraging distances are Gaussian distributed about the nest with a standard deviation σ_c. We take $\sigma_c = \sigma_f = 2$ km, as twice the mean foraging distance of *V. velutina*, accounting for the fact that interaction occur when foraging from two nests locations overlap. C_i therefore measures the intersection between foraging from nest i with foraging from all other nests.

The environmental suitability at a given site is based on a local (Gaussian) average over all nearby locations of the habitat type, as captured by the parameter *Terrain*.

$$E_i = \xi \sum_{j} exp\left(-\frac{\Delta_{ij}^2}{2\sigma_f^2}\right).Terrain_j . \quad (2)$$

Here σ_f =1 km, the mean foraging distance of *V. velutina*, such that the environmental suitability is the weighted average across all habitats that are likely to be visited. ξ is a normalising parameter, which ensures the sum of the Gaussians is equal to one. *Terrain* measures the suitability of the local habitat and is defined at every point, j in the landscape. The terrain-specific values for each location are determined using the Corine Land Cover data [4] and using the terrain preferences recorded from the French national nest data [5] to determine the appropriate weightings for each terrain-type Table S2. Whilst the Corine

dataset is highly detailed and had many categories of terrain type, we aggregate these into 5 broad classifications.

**Habitat Type**	***Terrain* value as specified by French national nest data (4)**
Artificial surfaces	1 (by default)
Agricultural areas	0.87
Forest & semi-natural areas	0.14
Wetlands	0.02
Other areas	0

Table S2: Habitat suitability as captured by the parameter *Terrain*, these preferences come from recording of nest locations from French national data[5,6].

We note that there is the potential for the observational values used to generate Table S2 to be biased by the ease of detecting nests in each of these habitat types; but given that the two major types (artificial and agricultural) that dominate the UK landscape have reasonably similar scores, this is unlikely to change the predicted behaviour.

With limited data on the effects of climate or latitude, we make the simplest assumption - that the number of successful nests decreases linearly with latitude, as captured by a parameter *f*. This was based on observations of the native hornet species, the European hornet (*Vespa Crabro*), which is at highest density in the south of England, with decreasing density going northwards; the most northerly location in which European hornets have been recorded is Yorkshire [7]. The function *f* is therefore assumed to decrease linearly from 1 at the latitude of Andernos-les-Bains to 0 at the northern most point of England. While there is clearly considerable uncertainty about both the quantitative and even the qualitative form of *f*, we note that it compares well with (4) which uses more detailed climatic variables to predict the probable spatial extent of *V. velutina*. In additional, given that *V. velutina* has already successfully colonised Northern Europe as far as Northern France and Belgium, we would expect them to be able to establish in Southern England. In the main text (Figure 2) we consider in some detail the impact of other assumptions for *f*, in particular how the value it takes at the site of invasion influences the subsequent dynamics.

To then determine how many successful queens (Q_i) would emerge from nest *i*, we bring together the estimated reproductive potential, the local carrying capacity, the effects of latitude and the density-dependent effects. The number of queens is taken from a Poisson distribution:

$$\mathrm{Q_i} = \mathrm{Poisson}\left(\frac{r.E_i\,.f(\mathrm{latitude}_i)}{1+\gamma C_i}\right). \quad (3)$$

The strength of the density-dependent effects (γ) and the naïve expected reproductive ratio (r) are inferred from the population dynamics of nests in Andernos-les-Bains [3].

Spatial spread. Once the number of successful queens is calculated, the associated nests are placed within the landscape based on the local terrain and the dispersal ability of the queens. In particular, the probability that a successful queen from location *i* creates a nest in location *j* in the subsequent year is proportional to:

$$P_{ij} = \exp\left(-\frac{\Delta_{ij}}{\mu}\right).Terrain_j \ .$$

Here Δ_{ij} captures the distance between the two locations, μ is the mean flight distance and, as above, *Terrain* measures the suitability of the local environment where the nest is to be built. Given that sea is classified as unsuitable habitat (*Terrain*=0), those queens born near the coast only choose a nest site from locations on land.

Here we have used exponentially distributed founding flights, where the mean flight distance μ = 28 km has been chosen to match a range of observed invasion wave-speeds: 67 km per year [8]; 75-82 km per year [9]. However, the precise value of μ, and the precise distribution of flights have a limited impact on many our findings. This is because much of our work is concerned with early establishment when density-dependence places a limited role; therefore, our results related to successful invasion are relatively insensitive to both the effects of density-dependent parameter (γ) and dispersal of queens (μ) which acts to mitigate density-dependence.

PARAMETER INFERENCE.

We use the temporal information from Andernos-les-Bains together with a non-spatial version of our model (equation (2)) to estimate parameters. A non-spatial model is valid for this dataset given the relatively small spatial scale of the Andernos-les-Bains region (only 20 km^2). The translation of the spatial model to a non-spatial version is considered below.

We assume that the total number of *V. velutina* colonies and hence primary nests (P_y) at the start of a given year (y) is Poisson distributed with a mean that is related to the number of successful secondary nests the year before (S_{y-1}) incorporating density dependence:

$$P_y = \text{Poisson}\left(\frac{rS_{y-1}}{1+\gamma\,\bar{C}\,S_{y-1}}\right) \qquad (3)$$

If left unchecked each of these primary nests later gives rise to a secondary nest, completing the life-cycle. Equation (3) is equivalent to equation (2) but assumes all nests are sufficiently close to experience competition as captured by the parameter $\bar{C}$:

$$\bar{C} = \overline{\exp\left(-\frac{\Delta_{ij}}{2\sigma_c^2}\right)}$$

where the average is taken over all points *i* and *j* in the Andernos-les-Bains region, chosen according to the *Terrain* weighting. Hence $\bar{C}$ measures the average density-dependent competition experienced by two randomly located nests in the Andernos-les-Bains region.

We then assume that active primary nests and active secondary nests (as well as inactive nests) are all discovered with given probabilities that are allowed to vary linearly over the period of observation. Only secondary nests that are not discovered (and destroyed) when active are considered successful and can contribute to the next year's foundresses. This leads to an inference problem involving: two demographic parameters, the intrinsic growth rate (r) and impact of density dependence (γ); five parameters that capture detection of different nest types; and the total number of primary nests at the start of each year, P_y. We

utilise a Metropolis Hastings MCMC (Markov Chain Monte Carlo) method to infer these parameters from the data in a Bayesian framework. Due to the complex interaction between detection, destruction and the invasion dynamics, simpler methods of calculating a population carrying capacity are not suitable.

For this non-spatial model, an endemic carrying-capacity (K) can be calculated for the entire Anderson-les-Bains region.

$$K_{AlB} = S_y^* = \frac{r-1}{\gamma \bar{C}} \tag{4}$$

We note that this carrying capacity scales with the reproduction ratio (r) and therefore will also scale with latitude in the UK model.

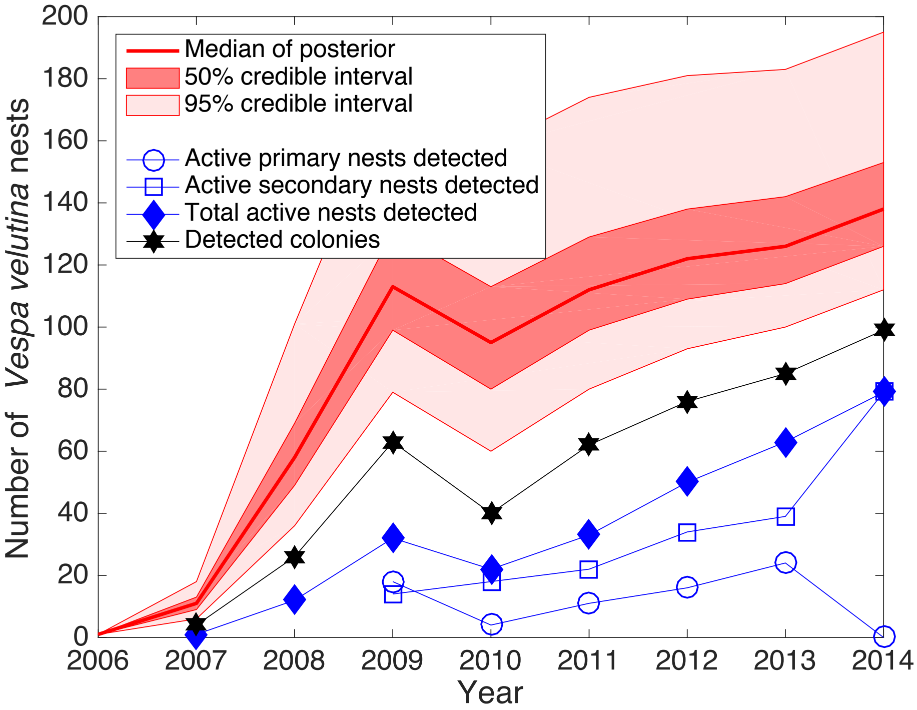

Figure S1. Time series of detected nests and posterior distributions for the total number of nests in the Andernos-les-Bains region. The number of active nests detected (and destroyed) which helps prevent the spread of *V. velutina* are shown in blue. A lower bound estimate of the total number of colonies is shown in black. While the red shaded areas give the 50% and 95% credible intervals for P_y (the estimated number of primary nests) in each year.

Figure S1 shows the available aggregate data from Andernos-les-Bains from 2007 to 2014, as presented in Table S1. The total number of active nests detected and destroyed (blue diamonds) play no role in the generation of next year's nests; we are therefore in the common situation whereby more intensive detection leads to more detection in the short-term, but greater control and therefore fewer nests in the longer-term. Some nests are discovered too late in the season, once the hornets have already left; these enable us to calculate the total number of hornet colonies detected in that year (black stars). Also shown in Figure S1 is the posterior distribution for the total number of nests in the area (P_y); we assume that the invasion starts with one (undetected) nest in 2006. The difference

between the red inferred curve and the blue diamonds is the number of colonies that are not destroyed and hence those that can generate nest year's nests.

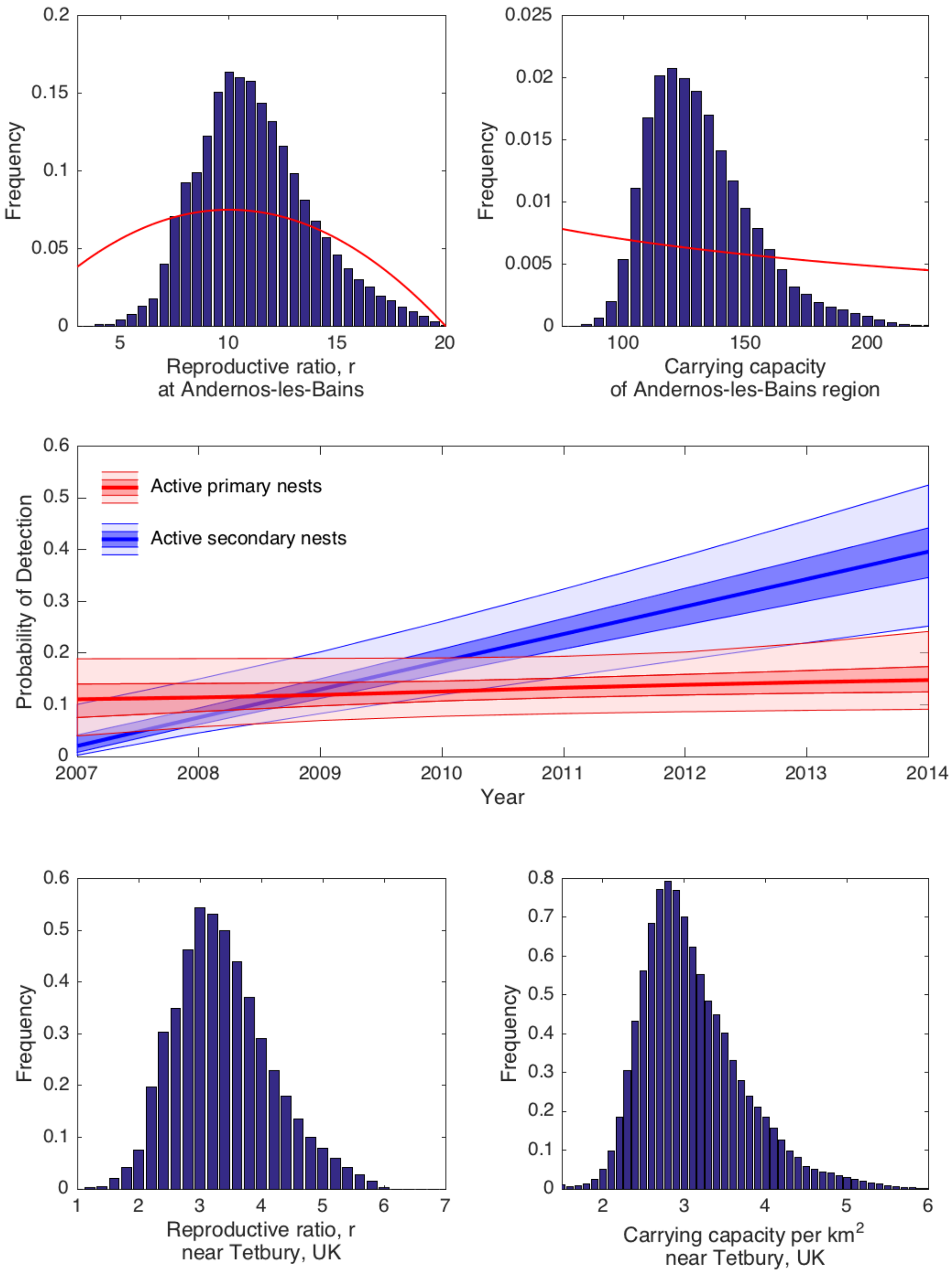

Figure S2. Results of Bayesian MCMC parameter inference using the nest detection data in the Andernos-les-Bains region. The top row shows the inferred distributions of the reproductive ratio, r, and the regional carrying capacity, K_{AlB}, together with the prior distributions (red). The centre graph shows how the detection probabilities of active primary (red) and secondary (blue) nests increases over the period; the shading gives the 95% and 50% credible intervals. The lower two graphs translate the Andernos-les-Bains parameters to those associated with the area around Tetbury, where the first UK nest was discovered.

Figure S2 shows the prior (red curve) and posterior (blue bars) distributions of two key demographic parameters (r and K_{AlB}; although the values of K_{AlB} are calculated from the fundamental parameters, r and γ), as well as the mean and credible intervals of the time-varying detection probabilities. Our inference shows that the reproductive ratio for the Andernos-les-Bains region has an expected value of 11.2 (95% CI 6.9 - 17.2); while the

carrying capacity of the 20 km^2 region is estimated at 131.7 (95% CI 100.1 – 184.6). Both detection probabilities are predicted to increase over time, with the greatest increase in detecting active secondary nests. By 2014, 15% (95% CI 9%-24%) of primary nests and 39% (95 CI 25%-52%) of secondary nests are discovered while active; so the overall chance of a nest being detected while active is 48% (95% CI 33%-61%).

The two lower graphs consider how these distributions translate to the UK in the locality of the first invasion near Tetbury in Somerset, taking into account both the latitude and the local terrain. The reproductive ratio, *r*, is reduced to 3.37 (95% CI 2.06 – 5.17) while the carrying capacity per km^2 is 3.10 (95% CI 2.17 – 4.65). It is the full posterior distribution associated with these modified values that is used in all the calculations within the main paper.

PROBABILISTIC CALCULATION OF INVASION DYNAMICS.

While Figures 1 and 3 in the main text are based on the results of multiple simulations, the maps and early dynamics shown in Figure 2 can be calculated at machine precision by using probabilistic arguments which we now outline.

We first need to calculate the probability that the 2015 invasion occurred at location *i* given that *V.velutina* were recorded at two locations r_1 and r_2 in 2016. This probability is given by:

$$P_i^{2015} = \zeta \; r \, E_i \, f(latitude_i) \times \left[\exp\left(-\frac{\Delta_{ir_1}}{\mu}\right).Terrain_{r_1}\right] \times \left[\exp\left(-\frac{\Delta_{ir_2}}{\mu}\right).Terrain_{r_2}\right].$$

Here, E_i is the effect of local habitat as defined in equation (2), the two terms in square brackets refer to dispersal to the recorded locations, and ζ is a normalising constant such that the probabilities sum to one. Given the linear dependence on *r* and the lack of density-dependent competition, the probabilistic position of invasion in 2015 (as shown in Figure 2A of the main text) is not affected by the parameter inference.

For the possible locations of undiscovered nests in 2016, we can again rely on the fact that there were no density-dependent interactions as we are assuming a single nest in 2015. Given the 2015 nest is assumed to be in location *i*, the probability that location *q* has an undiscovered nest in 2016 is:

$$P_{q|i}^{2016} = r \, E_i \, f(latitude_i) \times \frac{\exp\left(-\frac{\Delta_{iq}}{\mu}\right).Terrain_q}{\sum_p \exp\left(-\frac{\Delta_{ip}}{\mu}\right).Terrain_p}.$$

To obtain the probability of an undiscovered nest in location *q* in 2016 (as plotted in Figure 2B), the above quantity is averaged over all locations *i* for the nest in year 2015 and over the entire posterior distribution for the reproductive potential *r*.

$$P_q^{2016} = \int Post(r) \times \sum_i P_i^{2015} \times P_{q|i}^{2016} \; dr$$

This process can then be extended forwards to 2017, although here we technically need to account for the density dependent competition between nests in 2016 (even though this effect is very small). The probability of finding nests at location *s* in 2017 is then given by:

$$P_{s|i}^{2017} = \sum_q \frac{r\, E_q\, f\left(latitude_q\right)}{1+\gamma C_{q|i}} P_{q|i}^{2016} \times \frac{\exp\left(-\frac{\Delta_{qs}}{\mu}\right).Terrain_s}{\sum_p \exp\left(-\frac{\Delta_{qp}}{\mu}\right).Terrain_p} \ ,$$

where

$$C_{q|i} \ = \sum_p P_{p|i}^{2016} \times exp\left(-\frac{\Delta_{pq}^2}{2\sigma_c^2}\right) .$$

Again, to generate Figure 2C, we need to average over all locations i for the nest in year 2015 and over the entire posterior distributions for the reproductive potential *r* and the density-dependence γ.

Figures 2D-2F in the main text are then generated by changing the linear function of latitude, *f*. In particular, we change the gradient of *f* to achieve the desired reduction in the expected number of nests produced by an average nest in year 2015.

EXTENSIONS AND UNCERTAINTIES

A model that can predict the likely future dynamics of the invasion of *V. velutina* in the UK is of substantial use to policy makers. However, good quality data on the behaviour of *V. velutina* in Europe is lacking, with the vast majority of data coming from sparse observations. Given the need for a predictive model, we have taken one of the most detailed observations of local dynamics (from the Andernos-les-Bains region) and extrapolated to the UK. Clearly this gives rise to multiple uncertainties which we now discuss.

Impacts of Climate. We have referred throughout the work to our uncertainty in how climate effects the success of *V. velutina*. Detailed work such as that of Villemant *et al.* (4) provides a spatial estimate of the potential range of *V. velutina*, but cannot inform the mechanism nor the likely dynamics. We have therefore taken a simple approach, in which the reproductive ratio scales linearly with latitude as a proxy for the effects of climate. In Figure 2 (main text), we investigated in some detail the uncertainty of the invasion dynamics to the assumed reproductive ratio. We now consider the longer-term dynamics as shown in Figure 1 (main text).

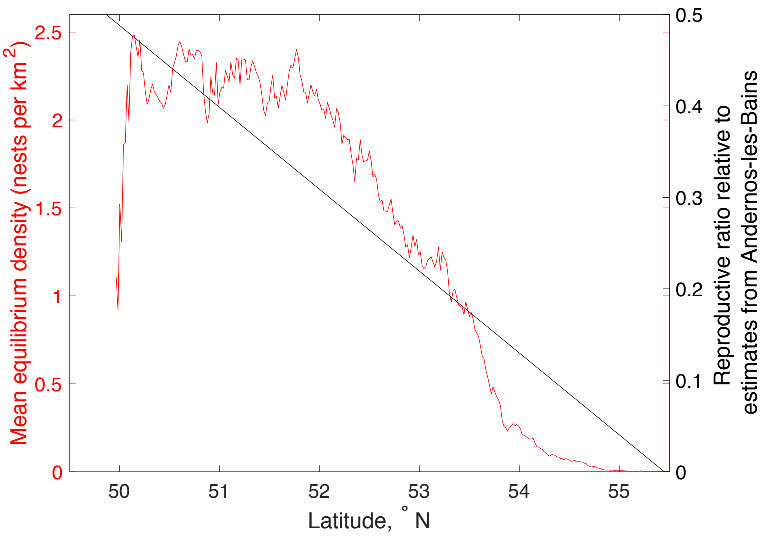

Figure S3 impact of latitude on the mean endemic density of nests and the reproductive ratio. Results from the full spatial model for the UK (as shown in Figure 1 of the main paper) are aggregated by latitude, showing the steady decline as we move further north. Results are from a thousand stochastic replicates with random draws from the posterior parameter distributions, and are taken 20 years after invasion when the population has reached equilibrium.

Figure S3 shows how the mean endemic density of nests (per km^2) varies with latitude (red curve) together with how the reproductive ratio declines (black line). In the absence of spatial factors and different terrain types, the equilibrium density is linearly related to the reproductive potential and hence the impact of latitude (see equation (4)). We note that while both show similar trends, the endemic density is far more non-linear; this is because it is influenced by both the local terrain, as well as the ability of queens to disperse to an area. For this reason, there is a 'tail' of nests at more northern latitudes, beyond the point where colonies can successfully sustain themselves (local reproductive ratio < 1). Similarly, there are also regions (near the coast) where there is more limited dispersal into the region due to the local geography. However, this graph provides a simple indication of how our assumptions about the impact of latitude (captured by the parameter f) are likely to influence the endemic distribution of nests.

Impacts of Food Sources. We have noted that *V. velutina*'s main food source is honey bees, which would suggest that the presence of apiaries may be a key factor in their success. Although the local density of apiaries could be included with the spatial simulation models of the UK, this would be confounded by the terrain data that is included. We know that apiaries are most likely to be near human habitation or agricultural areas, therefore disentangling the two effects is difficult. In addition, the data from Andernos-les-Bains (2) and on terrain preferences [5] does not allow us to distinguish between terrain and proximity to a plentiful supply of prey in the form of apiaries.

Wave Speed. The dispersal of queens to establish new nests is determined by fitting to observed wave-speeds of the invasion in France (7,8). Throughout we have assumed that this is a homogeneous exponentially-distributed dispersal, but other forms could be made to fit the data. An exponential was chosen as it provides a mix of predominately short-range dispersal with the potential for longer-range jumps. The spatial homogeneity assumption implies that neither habitat nor climate affect the dispersal of new queens, hence dispersal occurs equally at all latitudes and is not affected by the presence of

mountainous habitat or large bodies of water. Without any evidence to the contrary, this is the simplest assumption.

These three particular examples of assumptions that underlie the mathematical model highlight our general lack of knowledge about the behaviour of this invading predator. Much more detailed entomological studies of *V. velutina* are therefore required (ideally in the European setting) if we are to both increase the accuracy of predictive models and assist in control or eradication.

References

1 Monceau, K., Bonnard, O. & Thiery, D. Vespa velutina: a new invasive predator of honeybees in Europe. *Journal of Pest Science* **87**, 1-16, doi:10.1007/s10340-013-0537-3 (2014).

2 Marris, G., Brown, M. & Cuthbertson, A. G. *GB Non-native Organism Risk Assessment for Vespa velutina nigrithorax*, <www.nonnativespecies.org> (2011).

3 Franklin, D. N. *et al.* Invasion dynamics of Asian hornet, *Vespa velutina* (Hymenoptera: Vespidae): A case study of a commune in south-west France. *Applied Entomology and Zoology* (accepted, 2016).

4 European Environment Agency. *Corine Land Cover 2006 raster data*, <http://www.eea.europa.eu/data-and-maps/data/corine-land-cover-2006-raster-3> (2014).

5 Villemant, C. *et al.* Predicting the invasion risk by the alien bee-hawking Yellow-legged hornet Vespa velutina nigrithorax across Europe and other continents with niche models. *Biological Conservation* **144**, 2142-2150 (2011).

6 Archer, M. Taxonomy, distribution and nesting biology of the Vespa bicolor group (Hym., Vespinae). *Entomologist's Monthly Magazine* **130**, 149-158 (1994).

7 Archer, M. E. *Vespa crabro Linnaeus,1758*, <http://www.bwars.com/index.php?q=wasp/vespidae/vespinae/vespa-crabro> (1997).

8 Jung, C. Spatial expansion of an invasive hornet, *Vespa velutina nigrithorax* Buysson (Hymenoptera: Vespidae) in Korea. *Korean Journal of Apiculture* **27**, 87 - 93 (2012).

9 Robinet, C., Suppo, C. & Darrouzet, E. Rapid spread of the invasive yellow-legged hornet in France: the role of human-mediated dispersal and the effects of control measures. *Journal of Applied Ecology* (2016).
